# Microleakage, penetration depth, and fluoride release of Embrace Wetbond, Denuseal, and Helioseal F Plus pit and fissure sealants: a comparative in vitro study

**DOI:** 10.1186/s12903-026-08419-y

**Published:** 2026-05-06

**Authors:** Heba Mohamed Mahmoud Deghid, Basma Elsayed Hamza Elboraey, Rasha Ibrahim Ramadan, Rabaa Mahmoud Aboubakr

**Affiliations:** Dental Public Health and Preventive Dentistry, Department of Pediatric Dentistry and Dental Public Health, Faculty of Dentistry, Mansoura University, Mansoura City, Egypt

**Keywords:** Hydrophilic sealants, Hydrophobic sealants, Microleakage, Sealant Penetration, Fluoride release

## Abstract

**Background:**

The clinical longevity of pit and fissure sealants is closely related to their physical and chemical properties. To address the moisture sensitivity of hydrophobic pit and fissure sealants, hydrophilic types were introduced; however, their performance is not yet fully understood.

**Objectives:**

The present study was carried out to compare and evaluate microleakage, penetration depth, and fluoride release of three different pit and fissure sealants.

**Methods:**

Thirty extracted permanent upper first premolars were randomly distributed to three groups: Embrace WetBond, Denuseal, and Helioseal F Plus, each with or without bonding application (*n* = 5). Microleakage and penetration depth were evaluated using dye penetration, stereomicroscope examination, and image analysis software. Fluoride release was measured over four weeks using the SPANDS colorimetric method. SPSS version 27.0 was used for statistical analysis, and significance was set at *P* < 0.05.

**Results:**

Bonding application did not significantly reduce microleakage or improve penetration depth for any of the tested materials (*p* > 0.05). Helioseal F Plus demonstrated significantly lower microleakage and greater penetration under both bonded and non-bonded conditions compared with Denuseal (*p* < 0.05). Embrace WetBond showed higher microleakage and lower penetration depth than Helioseal F Plus. All tested pit and fissure sealants exhibited a pronounced initial fluoride release followed by a significant decline over time (*p* < 0.001). Embrace WetBond consistently gave the highest fluoride values among the three materials from Week 1 onward (*p* < 0.05).

**Conclusion:**

Helioseal F Plus demonstrated superior sealing ability with minimal microleakage and satisfactory penetration. Embrace WetBond exhibited enhanced fluoride release patterns.

**Supplementary Information:**

The online version contains supplementary material available at 10.1186/s12903-026-08419-y.

## Introduction

Caries is one of the main health problems worldwide, affecting both adults and children. The primary reason for the increasing incidence of dental caries is a diet rich in sugary foods and carbohydrates. The occlusal surface has nearly 60% of all caries lesions in the permanent dentition of children and adolescents [1]. Pit and fissure sealant (PFS) has been widely recognized as an effective preventive method for inhibiting caries formation on occlusal surfaces by creating a physical barrier that isolates highly susceptible fissures from the oral environment [2]. Despite their proven effectiveness, PFS failure may occur, and one of the main reasons for this failure is microleakage that allows the buildup of bacteria, nutrients, and acidic metabolic byproducts due to the inability to isolate pits and fissures [3]. 

An ideal PFS material possesses several crucial characteristics, such as retention, resistance to abrasion and wear, and the ability to seal and penetrate deeply into pits and fissures. Failure of the PFS can occur when the interface between the tooth and the material is compromised due to insufficient penetration into deep fissures. As moisture control in pediatric patients represents a significant clinical challenge and is a key determinant of preventive treatment success, hydrophilic sealants have been introduced to improve clinical handling under suboptimal isolation conditions [4]. Teeth that were sealed soon after eruption tend to remain free of cavities for much longer than those that are not sealed. Therefore, fluoride-releasing components are frequently incorporated into PFS formulations to enhance remineralization and provide antibacterial properties, leading to an advanced preventive effect [5, 6]. 

Factors that can affect fluoride release from PFS include material composition, fluoride recharge capacity, and ambient conditions. The speed and duration of ion release are influenced by the oral environment, pH variations, and topical fluoride agent contact. Therefore, the long-term therapeutic efficacy of PFS is determined by the balance between mechanical durability, fluoride release capacity, and the possibility of recharge under oral circumstances [7, 8]. 

Recent evidence suggests that the clinical success of PFS is increasingly dependent on the chemical nature of the resin and its interaction with the tooth surface. A systematic review by Gheidari et al. (2026) [9] concluded that while hydrophilic and hydrophobic sealants perform similarly in terms of long-term caries development and retention, hydrophobic sealants often exhibit superior penetration capabilities in controlled settings. This is complemented by recent in vitro findings suggesting that lower-viscosity materials, such as those found in moisture-tolerant systems like Embrace WetBond, may improve resin tag length and marginal adaptation, thereby reducing microleakage [10]. Furthermore, while some research advocates the routine use of adhesive systems to significantly minimize marginal gaps and enhance sealing [11], other studies emphasize that moisture-tolerant resin-based PFS can offer reliable clinical outcomes in cases where strict moisture control is challenging to achieve [9, 12]. 

Despite the continuous development of new PFS materials, there is still limited laboratory-based evidence comparing the performance of hydrophilic and hydrophobic sealants under standardized conditions [3, 13]. Most previous studies have evaluated individual properties such as microleakage or fluoride release separately, with limited data addressing these properties collectively [14–16]. In addition, the use of bonding agents has been suggested to enhance sealant retention and reduce microleakage; however, their effectiveness remains controversial and may depend on the type of sealant and application protocol [17–19]. Therefore, the present study aimed to evaluate the microleakage, sealant penetration depth, and fluoride release of three commercially available pit and fissure sealants (PFSs) representing different material categories (hydrophilic and hydrophobic systems), while also assessing the influence of bonding agent application under standardized in vitro conditions, to provide evidence that may assist in guiding clinical selection of the most suitable PFS material.

## Methods

### Study design

In this in vitro investigation, three different commercially available PFSs were evaluated for their microleakage, penetration depth, and fluoride release capacity.

### Ethical consideration

The study proposal was submitted to the Ethics Committee at the Faculty of Dentistry, Mansoura University (ethical approval # M0109024PP). Extracted teeth were collected from the Department of Oral and Maxillofacial Surgery at the Faculty of Dentistry, Mansoura University. This in-vitro study was conducted on extracted teeth according to the Declaration of Helsinki. Written informed consents were obtained from the patients to use their extracted teeth for research purposes after explaining the study aim.

### Sample size calculation

Using G*Power (version 3.1.9.7), sample size calculations were performed. For microleakage, based on Priscilla et al. (2023) [13] (effect size = 2.21, alpha = 0.05, and power = 90%), a minimum of six specimens was required; this was increased to ten per group (to account for potential specimen loss during thermocycling and to ensure robust subgroup analysis). Each group (10 teeth) was subdivided into two subgroups (5 teeth each) regarding the utilization of bonding agents. For fluoride release, based on Fita K et al. (2021) [14] (effect size = 2.21, alpha = 0.05, and power = 95%), ten specimens were used per group.

### Microleakage and sealant penetration depth evaluation

#### Sample preparation

Thirty recently extracted human upper permanent premolars, free from developmental defects, caries, or cracks, were selected, cleaned, and kept in saline. The crowns were embedded in self-cured acrylic resin with occlusal surfaces exposed, after root separation at the cement-enamel junction [13]. 

#### Grouping of samples

According to the type of PFS, the selected teeth were randomly allocated into three main groups (*n* = 10):

- Group I: Embrace WetBond (Pulpdent, USA)
- Group II: Denuseal (HDI Co., Ltd, South Korea)
- Group III: Helioseal F Plus (Ivoclar Vivadent, Liechtenstein)

Based on the application of the bonding agent, each group was subdivided into two subgroups (*n* = 5).

#### Surface preparation

Each tooth was treated with a 37% phosphoric acid gel for 20 s, then thoroughly rinsed with water and dried with air. In a hydrophilic PFS (Embrace WetBond), the enamel was gently dried and left slightly moist with a shiny appearance in accordance with the manufacturer’s instructions [15]. For both hydrophobic PFSs (Denuseal and Helioseal F Plus), enamel surfaces were dried with oil-free air for 10 s, until a frosty white appearance was achieved [16]. 

#### Bonding protocol

In the subgroups where a bonding agent was used, a universal bonding agent (3 M Single Bond Universal) was used in etch-and-rinse mode after phosphoric acid application according to the manufacturer’s instructions. This approach was specifically chosen because the etch-and-rinse technique is regarded as the gold standard for achieving optimal enamel bonding, which was essential for the present comparative evaluation of PFS microleakage [20]. The adhesive was actively applied, gently air-thinned, and light-cured using an LED light-curing unit. In the non-bonded subgroups, PFSs were applied directly after etching, without adhesive, according to the manufacturer’s instructions [12, 16]. 

### Pit and fissure sealant application

Following the manufacturer’s guidelines, each PFS was applied to the pits and fissures. The sealants were allowed to flow into the fissures before using the light-cure for the recommended curing time. After curing, the surfaces were checked to ensure complete coverage of the pits and fissures [12, 16]. 

### Thermocycling

The sealed teeth were subjected to thermocycling by passing through a hot bath at 55 °C and a cold bath at 5 °C for a total of 500 cycles, with a 60-second soak in every bath [9, 21]. The selected thermocycling protocol (500 cycles) is widely used in in-vitro studies to simulate thermal stresses in the oral environment and approximate short-term clinical aging conditions [22]. 

### Dye penetration

The methylene blue dye immersion technique was selected for this study as it provides a sensitive and reproducible method to detect microleakage and assess the sealing ability of the tested PFSs under stimulated clinical conditions [23, 24]. In this technique, the teeth were painted with layers of nail polish (six different colors to distinguish between groups), leaving only the PFSs and a 1 mm margin around them exposed. The teeth were immersed in a 1% methylene blue dye solution at room temperature for 24 h [25]. 

### Sectioning and microscopic examination

The teeth were gently brushed to remove excess dye. The teeth were sectioned buccolingually along the sealant using a diamond disk. One representative section of each tooth was examined under a stereomicroscope (SZ2-ILST, Olympus Corp, Tokyo, Japan) at 40x magnification and photographed using a digital camera [13]. The images were analyzed using IS Capture image analysis software (ToupTek Photonics, Zhejiang, China) [26]. Using one buccolingual section per tooth is a common protocol in many in-vitro microleakage studies [27, 28]. It was not assumed that microleakage or penetration is uniform throughout the entire fissure system. Consequently, the results represent a ‘snapshot’ of the seal, and assessing multiple sections might have revealed variations in the true seal of the specimens analyzed [11, 29]. 

### Evaluation of microleakage and penetration depth: [30]

A blinded examiner assessed microleakage and sealant penetration depth on sectioned specimens using a stereomicroscope (40×) and digital image analysis software. The lengths of dye penetration (L1, L2 in µm) and the corresponding tooth-sealant interface lengths (L3, L4 in µm) are used to calculate the microleakage proportion.

$$Microleakage\;proportion\:=\:\frac{\boldsymbol{L}1+\boldsymbol{L}2}{\boldsymbol{L}3+\boldsymbol{L}4}$$

The sealant penetration capability was determined as the proportion of the filled area by the sealant (C1 in µm^2^) relative to the whole area of the fissure that the sealant should be filled (C1 + C2), where (C2 in µm^2^) is the unfilled area of the sealant.

$$Penetration\;proportion \:=\:\frac{\boldsymbol{C}1}{\boldsymbol{C}1+\boldsymbol{C}2}$$

**Fig. 1:**
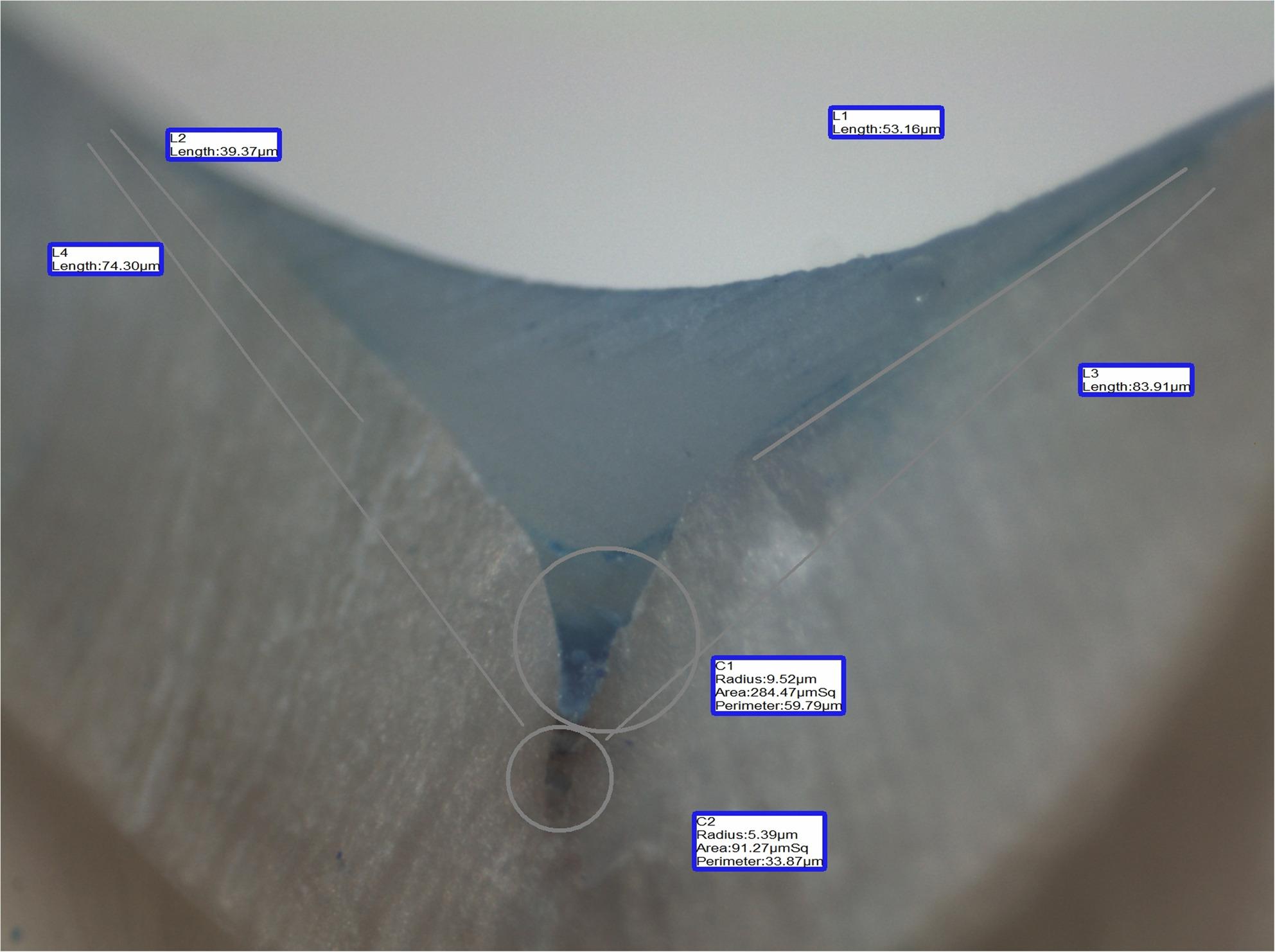
Representative microphotograph of sectioned specimens showing the measurement of microleakage (L1–L4) and sealant penetration (C1, C2) using a stereomicroscope and digital image-analysis software

Both microleakage and penetration depth proportions are unitless, allowing standardized, quantitative evaluation of sealing ability and penetration depth. (Fig. 1)

### Fluoride release analysis using SPANDS colorimetric method

#### Specimen preparation

Standardized disc-shaped specimens were fabricated from each tested PFS using custom Teflon molds (5 mm diameter × 2 mm thickness). After removal of excess material using a glass slide to ensure flat and uniform surfaces, specimens were light-cured according to the manufacturer’s instructions. A total of ten specimens per material were prepared and individually weighed using an analytical balance to ensure consistency [31]. 

### Storage conditions and immersion protocol

Each specimen was individually immersed in 10 mL of artificial saliva in sterile polypropylene screw-cap tubes. Polypropylene containers were selected to minimize fluoride adsorption and prevent interaction with the container walls [32, 33]. The artificial saliva was prepared using NaCl (0.4 g/L), KCl (0.4 g/L), CaCl₂ (0.9 g/L), and NaH₂PO₄ (0.7 g/L) dissolved in distilled water, with pH adjusted to 6.8 to simulate oral conditions [34]. Specimens were incubated at 37 °C under static conditions throughout the experimental period to mimic intraoral temperature [35]. The pH of the storage medium was monitored using a pH meter (VWR Symphony, USA) at each evaluation interval and remained stable, ensuring that fluoride release was not influenced by pH fluctuations [36]. 

### Fluoride release measurement and sampling intervals

Fluoride release was evaluated at standardized time intervals: 1 day, 1 week, 2 weeks, 3 weeks, and 4 weeks. At each time point, 5 mL aliquots of the storage solution were collected for analysis, and the total volume of the storage medium was maintained constant throughout the study period. The selected evaluation period was based on established evidence demonstrating an initial high fluoride release (burst effect) within the first 24–72 h, followed by a gradual decline and stabilization after a one-month period [37–39]. 

### Spectrophotometric analysis using SPANDS colorimetric method

Fluoride concentration was determined using the SPANDS colorimetric method in conjunction with a UV–visible spectrophotometer (Lambda 365, PerkinElmer) [40]. This method is based on the reaction between fluoride ions and a zirconium–dye complex, where fluoride ions form a stable hexafluorozirconate complex (ZrF₆²⁻), resulting in a proportional decrease in color intensity [40]. The SPANDS reagent consisted of sodium 2-(p-sulfophenylazo)-1,8-dihydroxy-3,6-naphthalene disulfonate and a zirconyl-acid reagent prepared from zirconyl chloride octahydrate dissolved in hydrochloric acid [40]. For analysis, 5 mL of the collected sample was mixed with 5 mL of freshly prepared SPANDS reagent. After allowing 1 min for complete color development at room temperature, absorbance was measured at 570 nm, corresponding to the maximum absorbance wavelength (λmax) of the complex [40]. The decrease in absorbance values (observed as negative absorbance changes) reflects the inverse relationship between fluoride concentration and color intensity due to the formation of the fluoride–zirconium complex in the SPANDS method.

### Calibration curve and quantification

**Fig. 2:**
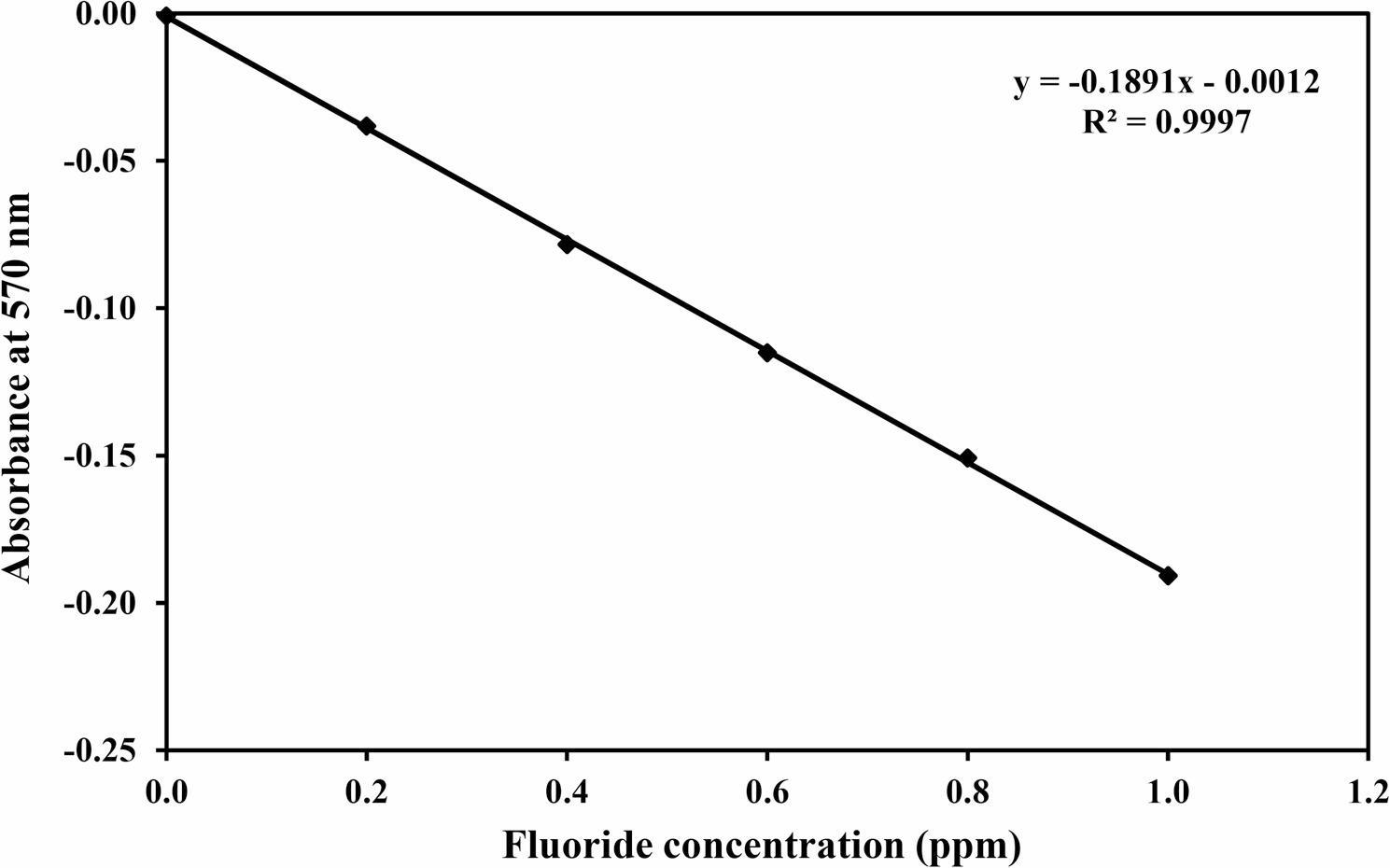
Calibration curve of fluoride standards (0–1.0 ppm) using the SPANDS colorimetric method, showing a strong inverse linear relationship between fluoride concentration and absorbance at 570 nm (*R*² = 0.9997)

A calibration curve was constructed using fluoride standard solutions in the range of 0–1.0 ppm (0, 0.2, 0.4, 0.6, 0.8, and 1.0 ppm) [40]. The calibration curve demonstrated excellent linearity (*R*² =0.9997), confirming the reliability of the method for quantitative analysis. Fluoride concentrations in the samples were calculated by interpolation from the calibration curve (Fig. 2).

### Method validation and analytical reliability

To ensure analytical precision, all measurements were performed in triplicate, and mean values were used for statistical analysis. The method demonstrated high repeatability, with relative standard deviation (RSD) values ranging from 0.11% to 0.54%. Each sample was analyzed in triplicate at each time point per specimen. Blank artificial saliva samples showed negligible absorbance at 570 nm, confirming the absence of interference from the storage medium or the polypropylene containers [40]. The SPANDS method is widely recognized for its sensitivity and specificity in fluoride determination in aqueous systems, making it suitable for evaluating fluoride release from dental materials [40]. 

### Statistical analysis of the data

Statistical analysis was performed using SPSS version 27.0 (IBM Corp., Armonk, NY, USA). The normality of data distribution for each outcome variable was independently assessed using the Shapiro-Wilk test.

#### Microleakage and penetration (non-normally distributed data)

Microleakage scores and sealant penetration depth did not follow normal distribution; therefore, these data are presented as Median and Interquartile Range (IQR). Comparisons between the bonded and non-bonded groups were performed using the Mann-Whitney U test. Comparisons among the three sealant types were conducted using the Kruskal-Wallis test followed by Dunn’s post hoc test for pairwise comparisons. In all instances, the level of statistical significance was set at *p* ≤ 0.05.

#### Fluoride release (normally distributed data)

Data for fluoride release followed a normal distribution and are presented as Mean ± Standard Deviation (SD). Intergroup comparisons were conducted using one-way ANOVA followed by Tukey’s post hoc test. For longitudinal analysis across the four weeks, repeated-measures ANOVA with Bonferroni-adjusted post hoc tests was utilized.

## Results

**Fig. 3:**
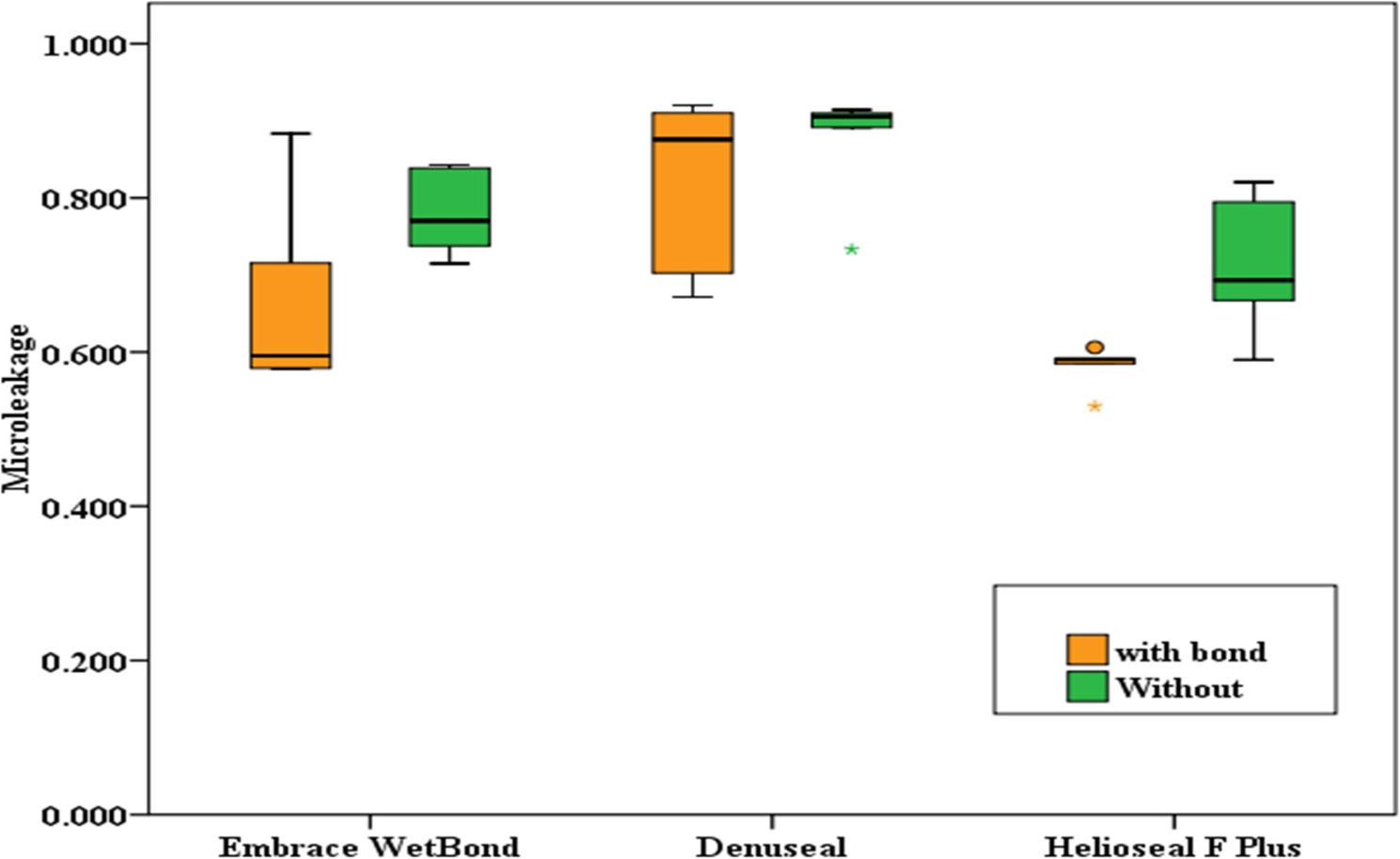
Intra-group Comparison of Microleakage (Effect of Bonding)

**Fig. 4:**
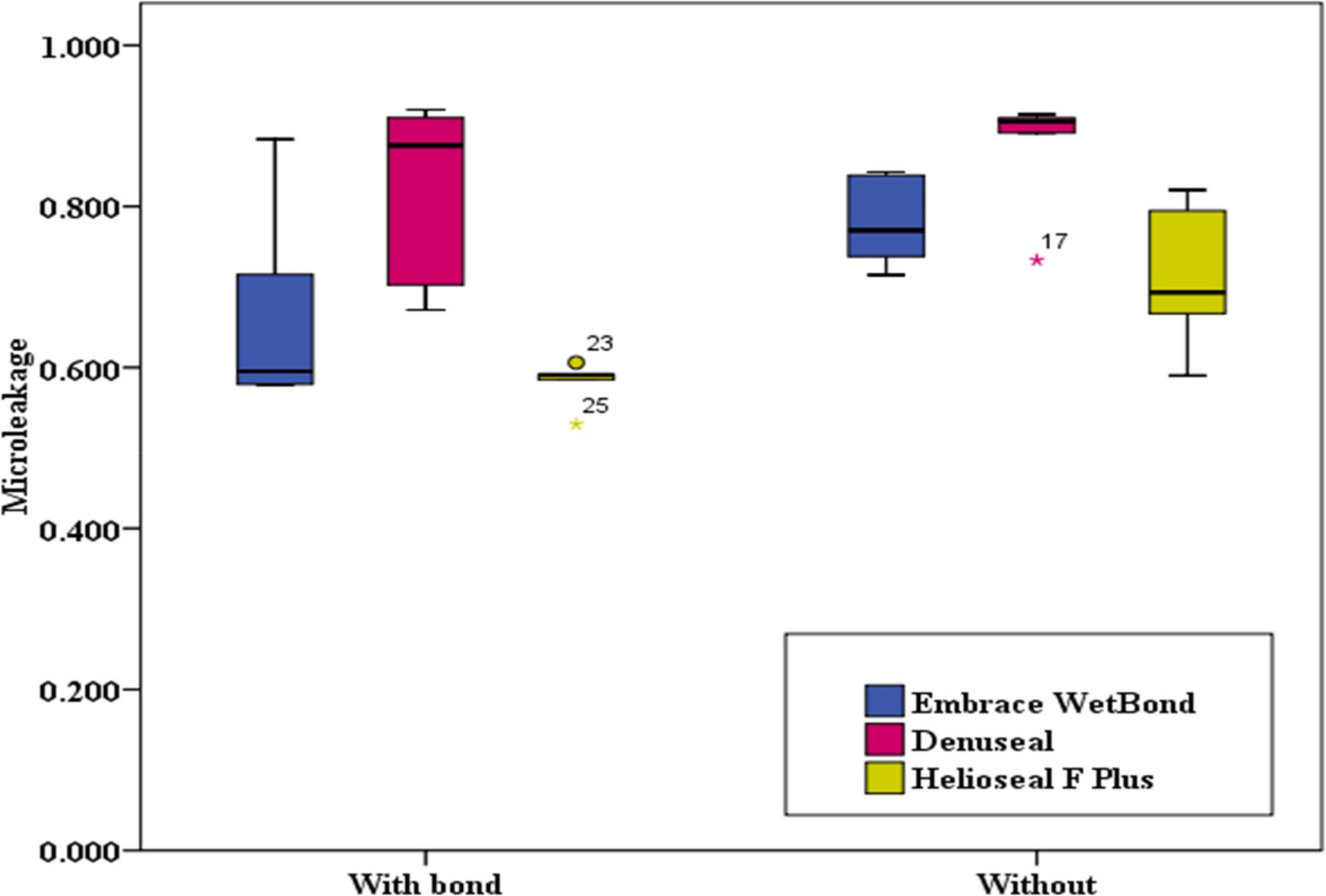
Inter-group Comparison of Microleakage (Difference between Materials)

Microleakage and penetration data were not normally distributed (Shapiro–Wilk test, *p* < 0.05), while fluoride release data followed a normal distribution (*p* > 0.05). Table 1 presents the microleakage scores across the three PFS groups (Embrace WetBond, Denuseal, and Helioseal F Plus) under bonded and non-bonded conditions. Concerning the effect of bonding, between the “with bond” and “without bond” subgroups, although no statistically significant difference was found for any of the materials (*p* > 0.05), Helioseal F Plus showed a trend toward reduced microleakage with bonding (*p* = 0.056). In both bonded (H = 6.72, *p* = 0.035) and non-bonded (H = 6.54, *p* = 0.038) conditions, between the three sealant materials, significant differences were observed. Post-hoc analysis revealed that Helioseal F Plus exhibited significantly lower microleakage compared to Denuseal (*p* = 0.011 in both conditions). Embrace WetBond showed intermediate values that did not differ significantly from the other two groups. (Figures 3 and 4)

**Table 1:** Comparison of Microleakage Among the Three Studied Pit and Fissure Sealants with and without Bonding

Sealant Group	The bonding Condition	Median (IQR)	Range	Test Statistic (*P*-Value)
Intra-group Comparison (Effect of Bonding)				
Embrace WetBond	With Bond (*n* = 5)	0.595 (0.579–0.715)	0.579–0.883	U = 6.00 (0.222)
	Without Bond (*n* = 5)	0.770 (0.738–0.838)	0.715–0.843	
Denuseal	With Bond (*n* = 5)	0.876 (0.702–0.910)	0.672–0.920	U = 10.00 (0.690)
	Without Bond (*n* = 5)	0.905 (0.812–0.912)	0.733–0.914	
Helioseal F Plus	With Bond (*n* = 5)	0.591 (0.585–0.591)	0.530–0.606	U = 3.00 (0.056)
	Without Bond (*n* = 5)	0.693 (0.667–0.795)	0.590–0.820	
Inter-group Comparison (Difference between Materials)				
With Bond	Embrace (*n* = 5)	0.595 (0.579–0.715)	0.579–0.883	H = 6.72 (0.035*)
	Denuseal (*n* = 5)	0.876 (0.702–0.910)	0.672–0.920	***(Pairwise: HF vs. D***, p = 0.011*)
	Helioseal F Plus (*n* = 5)	0.591 (0.585–0.591)	0.530–0.606	
Without Bond	Embrace (*n* = 5)	0.770 (0.738–0.838)	0.715–0.843	H = 6.54 (0.038*)
	Denuseal (*n* = 5)	0.905 (0.812–0.912)	0.733–0.914	***(Pairwise: HF vs. D***, ***p =*** 0.011*)
	Helioseal F Plus (*n* = 5)	0.693 (0.667–0.795)	0.590–0.820	

Pairwise comparisons were performed using Dunn's Post Hoc Test

Non-significant pairwise results are omitted for brevity

Microleakage = dye penetration depth/ total fissure length (µm)

*IQR* Interquartile Range, *U* Mann-Whitney U, *H* Kruskal-Wallis Test, *EW* Embrace WetBond, *D* Denuseal, *HF* Helioseal F Plus

*Statistically significant at *p* ≤ 0.05

**Fig. 5:**
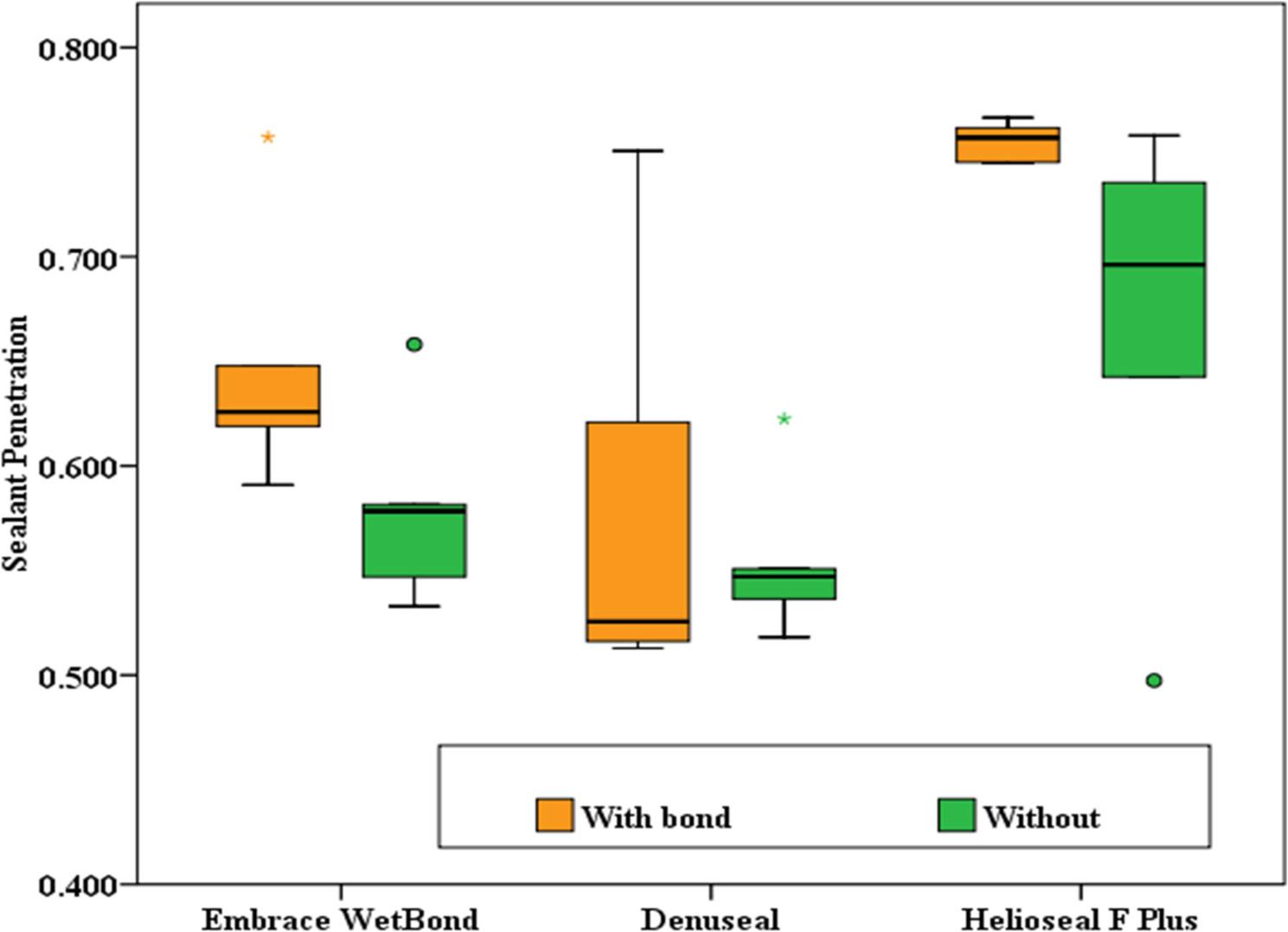
Intra-group Comparison of Sealant Penetration (Effect of Bonding)

**Fig. 6:**
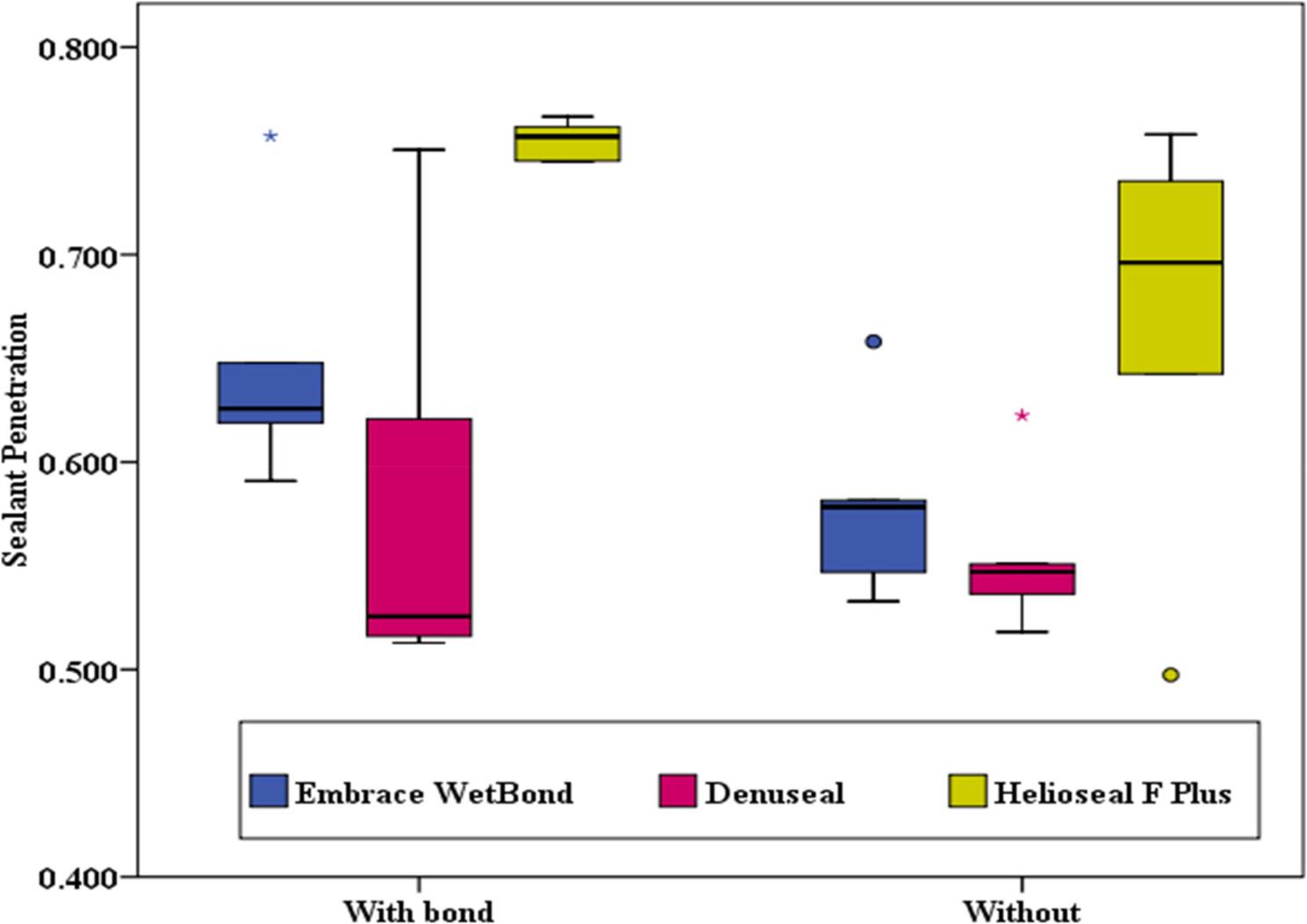
Inter-group Comparison of Sealant Penetration (Difference between Materials)

Table 2 summarizes the sealant penetration depth. For the Bonding effect, the application of a bonding agent did not yield a statistically significant improvement in penetration depth for any group (*p* > 0.05). However, the comparison of three tested materials yielded a significant difference in penetration among the bonded groups (H = 6.98, *p* = 0.031). Helioseal F Plus demonstrated significantly deeper penetration compared to Denuseal (*p* = 0.009). While in the absence of a bonding agent among the three materials, no significant differences in penetration depth were found (H = 3.26, *p* = 0.196). (Figures 5 and 6)

**Table 2:** Comparison of Sealant Penetration Depth Among the Three Studied Sealants with and without Bonding

Sealant Group	Condition	Median (IQR)	Range	Test Statistic (*P*-Value)
Intra-group Comparison (Effect of Bonding)				
Embrace WetBond	With Bond (*n* = 5)	0.626 (0.619–0.648)	0.591–0.757	U = 4.00 (0.095)
	Without Bond (*n* = 5)	0.578 (0.547–0.582)	0.533–0.658	
Denuseal (*n* = 5)	With Bond (*n* = 5)	0.526 (0.516–0.621)	0.513–0.751	U = 10.00 (0.690)
	Without Bond (*n* = 5)	0.547 (0.536–0.551)	0.518–0.622	
Helioseal F Plus (*n* = 5)	With Bond (*n* = 5)	0.757 (0.745–0.762)	0.745–0.767	U = 3.00 (0.056)
	Without Bond (*n* = 5)	0.696 (0.643–0.735)	0.497–0.758	
Inter-group Comparison (Difference between Materials)				
With Bond	Embrace (*n* = 5)	0.626 (0.619–0.648)	0.591–0.757	H = 6.98 (0.031*)
	Denuseal (*n* = 5)	0.526 (0.516–0.621)	0.513–0.751	*(****Pairwise: HF vs****. ****D***, p = 0.009*)
	Helioseal F Plus (*n* = 5)	0.757 (0.745–0.762)	0.745–0.767	
Without Bond	Embrace (*n* = 5)	0.578 (0.547–0.582)	0.533–0.658	**H = 3.26** **(** **0.196)**
	Denuseal (*n* = 5)	0.547 (0.536–0.551)	0.518–0.622	
	Helioseal F Plus (*n* = 5)	0.696 (0.643–0.735)	0.497–0.758	

Pairwise comparisons were performed using Dunn's Post Hoc Test

Non-significant pairwise results are omitted for brevity

Penetration = filled sealant area/ (filled area+ non filled area) (µm^2^)

*IQR* Interquartile Range, *U* Mann-Whitney U, *H* Kruskal-Wallis Test, *EW* Embrace WetBond, *D* Denuseal, *HF* Helioseal F Plus

*Statistically significant at *p* ≤ 0.05

**Fig. 7:**
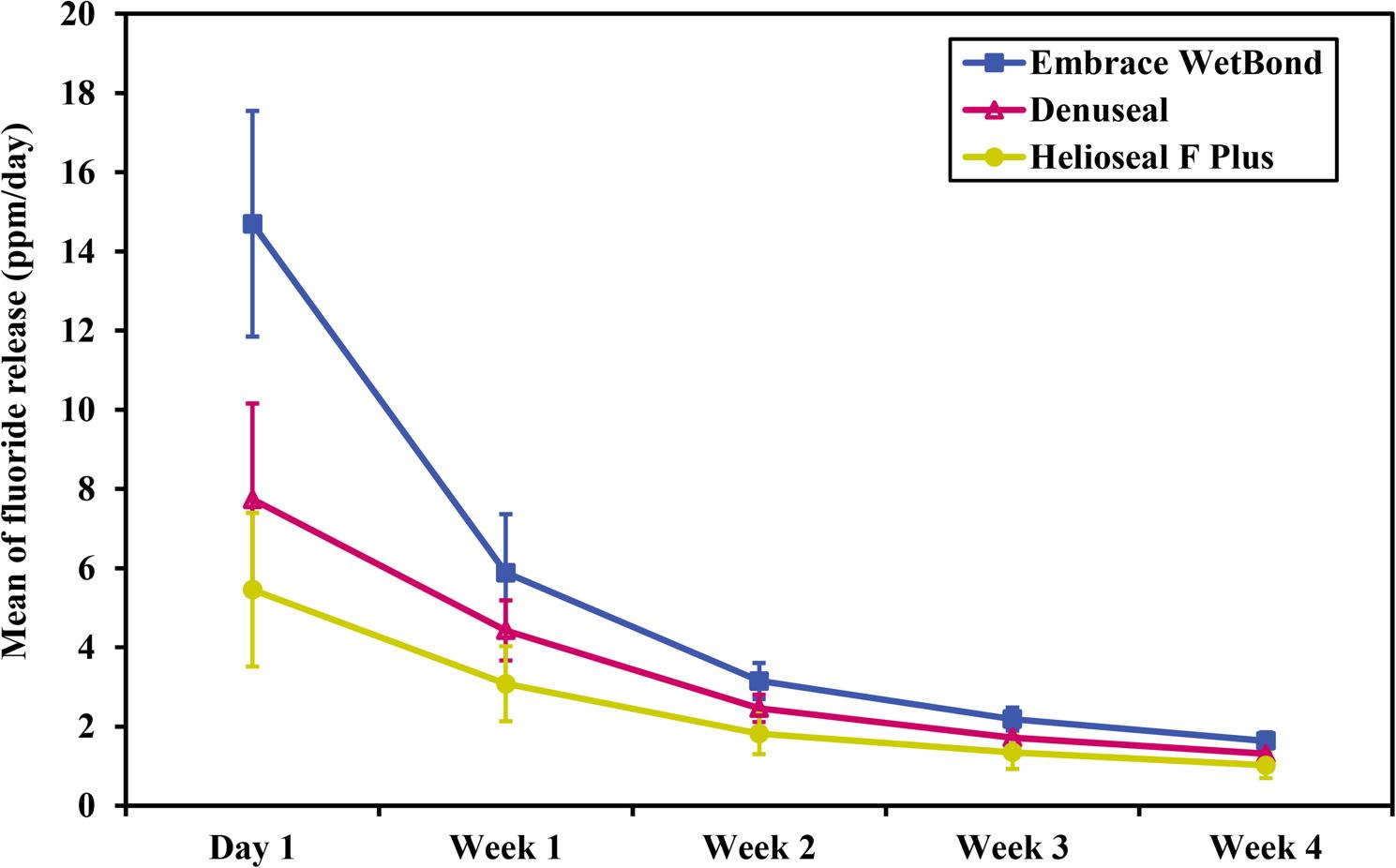
Time-dependent fluoride release (ppm) of the three studied sealants

Table 3 and Fig. 7 illustrate the fluoride release (ppm) for the three sealant groups over four weeks. All sealant materials exhibited the highest fluoride release on the first day. Embrace WetBond demonstrated the highest initial burst (14.70 \pm 2.85 ppm), which was significantly higher than both Denuseal (7.74 \pm 2.42 ppm, *p* < 0.001) and Helioseal F Plus (5.46 \pm 1.94 ppm, *p* < 0.001).

**Table 3:** Time-Dependent Fluoride Release (ppm) of the Three Studied Pit and Fissure Sealants

Time point	Embrace WetBond (*n* = 10)	Denuseal (*n* = 10)	Helioseal F Plus (*n* = 10)	F1	*p*-value
Day 1				**39.150**	< 0.001*
Mean ± SD	**14.70 ± 2.85**	7.74 ± 2.42	5.46 ± 1.94		
Min–Max	10.21–18.28	4.53–13.40	3.14–9.21		
Pairwise (Tukey)	EW–D < 0.001*	EW–HF < 0.001*	D–HF = 0.110		
Week 1				**16.162**	< 0.001*
Mean ± SD	**5.89 ± 1.47**	4.43 ± 0.76	3.08 ± 0.95		
Min–Max	3.30–7.81	3.20–6.00	2.00–5.10		
Pairwise (Tukey)	EW–D = 0.017*	EW–HF < 0.001*	D–HF = 0.029*		
Week 2				**22.277**	< 0.001*
Mean ± SD	**3.15 ± 0.46**	2.46 ± 0.34	1.82 ± 0.52		
Min–Max	2.55–4.03	1.90–3.10	1.30–3.12		
Pairwise (Tukey)	EW–D = 0.005*	EW–HF < 0.001*	D–HF = 0.009*		
Week 3				**16.553**	< 0.001*
Mean ± SD	**2.19 ± 0.29**	1.72 ± 0.26	1.35 ± 0.42		
Min–Max	1.70–2.60	1.30–2.20	1.10–2.50		
Pairwise (Tukey)	EW–D = 0.009*	EW–HF < 0.001*	D–HF = 0.044*		
Week 4				**16.099**	< 0.001*
Mean ± SD	**1.64 ± 0.21**	1.31 ± 0.19	1.02 ± 0.32		
Min–Max	1.38–1.95	1.00–1.65	0.80–1.90		
Pairwise (Tukey)	EW–D = 0.014*	EW–HF < 0.001*	D–HF = 0.035*		
Repeated measures ANOVA (F2, P)	**(134.770)** < 0.001*	**(75.463)** < 0.001*	**(58.884)** < 0.001*		

F1: One - way ANOVA for comparison between materials at each time point

F2: Repeated measures ANOVA was used for within-material comparisons across time, followed by Bonferroni-adjusted post hoc tests

Tukey’s post hoc test was applied for pairwise comparisons between materials

*Statistically significant at *p* ≤ 0.05

Fluoride release measured in ppm

*EW* Embrace WetBond, *D* Denuseal, *HF* Helioseal F Plus

At this initial stage, there was no significant difference between Denuseal and Helioseal F Plus (*p* = 0.110). For long-term release (Weeks 1–4), a consistent decline in fluoride release was observed for all groups over time (*p* < 0.001 for within-group comparisons). By Week 1, fluoride release levels decreased by approximately 40–60%. Unlike Day 1, the differences between all three materials became statistically significant at every subsequent time point (*p* < 0.05 for all pairwise comparisons).

Final levels (Week 4): By the end of the study, Embrace WetBond maintained the highest release (1.64 \pm 0.21 ppm), followed by Denuseal (1.31 \pm 0.19 ppm) and Helioseal F Plus (1.02 \pm 0.32 ppm).

Spearman correlation analysis revealed no overall statistically significant correlations between fluoride release and either microleakage or sealant penetration across all of tested materials with and without bond and time points (*p* > 0.05). However, isolated statistically significant correlations were observed, including Embrace WetBond without bond from Week 1 to Week 4 (*p* = 0.037) and Denuseal without bond at Week 3 (*p* < 0.001) (Supplementary File 1, Table 4).

## Discussion

The present in vitro study provides a comprehensive comparison of microleakage, penetration depth, and fluoride release among three pit and fissure sealants under standardized conditions. The findings demonstrate that sealing ability is primarily governed by the physical properties of the material rather than its fluoride-releasing capacity. Specifically, Helioseal F Plus exhibited superior marginal integrity and deeper fissure penetration, whereas Embrace WetBond demonstrated the highest fluoride release. These results highlight that these properties are complementary but not directly correlated.

### Effect of bonding agent application

The application of a bonding agent did not significantly influence microleakage or penetration depth for any of the tested materials. This finding suggests that adequate enamel etching alone may provide sufficient micromechanical retention under optimal conditions, reducing the additional benefit of adhesive application. These results matched with several studies that have reported bonding agents did not enhance sealing performance when proper etching and isolation are achieved [12, 41–45]. However, conflicting evidence exists, as Askarizadeh et al. [46], Meller et al. [47], and Nahvi et al. [48] have demonstrated improved marginal adaptation and reduced microleakage with the use of bonding agents, particularly under conditions of moisture contamination. This discrepancy may be attributed to differences in material composition, adhesive systems, and experimental protocols. Therefore, the effectiveness of bonding agents appears to be material-dependent and technique-sensitive, rather than universally beneficial.

### Microleakage and penetration depth

The superior performance of Helioseal F Plus in terms of reduced microleakage and increased penetration depth can be attributed to its lower viscosity and optimized filler composition, which facilitate deeper infiltration into narrow fissures and improved adaptation to enamel surfaces. In contrast, Denuseal exhibited higher microleakage and reduced penetration, possibly due to increased viscosity associated with its filler content, which may hinder its ability to flow effectively into complex fissure morphology. Embrace WetBond demonstrated intermediate performance, reflecting a balance between its hydrophilic formulation and flow characteristics. While its moisture tolerance may enhance handling under clinical conditions, it may not fully compensate for its comparatively lower penetration ability under controlled in vitro conditions.

These findings are consistent with Gawali et al. [4], Ku et al. [15], and Anika et al. [49], who indicated that hydrophobic sealants generally achieve superior penetration and marginal adaptation in dry conditions. In contrast, hydrophilic materials may perform better in moisture-compromised environments. It is important to note that these differences in reporting microleakage and penetration outcomes across studies could be attributed to variations in methodologies, including magnification, sectioning protocols, and image analysis techniques [3, 50–52]. Therefore, methodological standardization remains essential for reliable comparison between studies.

### Fluoride release behavior

All tested sealants exhibited a characteristic initial fluoride “burst effect”, followed by a gradual decline over time. Embrace WetBond demonstrated the highest initial and cumulative fluoride release, which may be attributed to its hydrophilic resin matrix and increased water sorption, facilitating ion diffusion through the polymer network. This release pattern is consistent with Şişmanoğlu [31] and Ito et al. [53], who reported that fluoride-containing materials release a large proportion of fluoride within the first 24–48 h, followed by a sustained but lower release phase. Such a release profile is considered beneficial, as the initial burst may enhance early enamel remineralization, while the sustained release contributes to long-term cariostatic effects. Despite the decline in fluoride release over time, the levels observed at later stages remained within the therapeutic range (0.03-1 ppm) reported in the literature for promoting remineralization and inhibiting demineralization [54, 55]. This suggests that fluoride-releasing sealants may provide ongoing preventive benefits beyond the immediate post-application period.

### Interrelationship between fluoride release and microleakage

An important finding of this study is the absence of a consistent correlation between fluoride release and microleakage. Although Embrace WetBond exhibited the highest fluoride release, it did not demonstrate superior sealing ability. Conversely, Helioseal F Plus showed minimal microleakage despite lower fluoride release. However, isolated statistically significant correlations were observed in specific subgroups. This observation indicates that microleakage is predominantly influenced by physical and mechanical properties, such as viscosity, wettability, and penetration depth, whereas fluoride release is governed by material composition and ion diffusion mechanisms. Similar findings have been reported in previous studies, confirming that fluoride release does not necessarily predict marginal integrity [35, 38, 39]. 

Nevertheless, fluoride release may play a protective adjunctive role in the presence of marginal gaps. Fluoride ions can diffuse into adjacent enamel, enhancing remineralization and reducing the risk of secondary caries even when some degree of microleakage is present. Therefore, these two properties should be considered complementary rather than interdependent, and optimal sealant performance should ideally combine effective marginal sealing with sustained fluoride release [37, 54]. 

### Clinical implications

From a clinical perspective, the findings of this study suggest that material selection should prioritize sealing ability and penetration characteristics, as these directly influence the prevention of microleakage and subsequent caries development. While fluoride release contributes to the preventive effect of sealants, it should be considered a secondary, supportive property rather than the primary determinant of clinical success. Hydrophilic sealants may offer advantages in situations where moisture control is challenging; however, under optimal isolation conditions, hydrophobic materials with superior flow characteristics may provide better long-term sealing performance.

### Strengths and limitations

The strengths of this study include a standardized experimental design, simultaneous evaluation of multiple clinically relevant outcomes, and the use of validated quantitative methods, including image analysis and spectrophotometry. However, several limitations should be acknowledged. As an in vitro study, the findings may not fully replicate clinical conditions due to the absence of dynamic oral factors such as salivary flow, pH fluctuations, enzymatic activity, and masticatory forces. In addition, the use of premolars instead of molars and inherent differences in enamel and fissure morphology may affect external validity. Evaluating a single buccolingual section per tooth may not fully capture the variability of microleakage and penetration across the fissure system, potentially limiting the comprehensiveness of the sealing assessment. Furthermore, the thermocycling protocol represents a short-term aging model that may not reflect long-term intraoral conditions. The relatively small sample size may also limit the generalizability of the findings and may increase the risk of a Type II error, particularly in relation to the non-significant findings observed for the bonding effect. Future studies should incorporate long-term clinical evaluations and more closely simulate intraoral conditions.

## Conclusion

Within the limitations of this in vitro study, sealant performance was primarily determined by material-dependent physical properties, particularly penetration and marginal adaptation, rather than fluoride release alone. Helioseal F Plus demonstrated superior sealing ability, while Embrace WetBond exhibited the highest fluoride release. The lack of correlation between these outcomes indicates that they are complementary but independent properties. The bonding application did not significantly influence performance under controlled conditions. Clinically, priority should be given to effective sealing, with fluoride release considered an adjunctive benefit. Further clinical studies are required to confirm these findings.

## Supplementary Information

Supplementary Material 1.

Supplementary Material 2.

## Data Availability

The data supporting the findings of this study are available from the corresponding author upon reasonable request.
