# Supplementary material for: Microleakage, penetration depth, and fluoride release of Embrace Wetbond, Denuseal, and Helioseal F Plus pit and fissure sealants: a comparative in vitro study
Source: BMC Oral Health. 2026 May 6;26:796. doi: 10.1186/s12903-026-08419-y (PMC13151319; doi:10.1186/s12903-026-08419-y)
Supplement: Supplementary file 1 — Supplementary Material 1. [file 12903_2026_8419_MOESM1_ESM.docx]

**Table 4. Spearman Correlation between Fluoride release with Microleakage and Sealant penetration in each group**

|  |  | **Sealant Group** |  | **Fluoride release (ppm/day)** | | | | |
| --- | --- | --- | --- | --- | --- | --- | --- | --- |
|  |  |  |  | **Day 1** | **Week 1** | **Week 2** | **Week 3** | **Week 4** |
| **Microleakage** | **With Bond** | **Embrace WetBond (n = 5)** | **r_s_** | -0.100 | 0.100 | 0.100 | -0.600 | -0.600 |
|  |  |  | **p** | 0.873 | 0.873 | 0.873 | 0.285 | 0.285 |
|  |  | **Denuseal (n = 5)** | **r_s_** | -0.100 | -0.100 | -0.100 | -0.100 | -0.100 |
|  |  |  | **p** | 0.873 | 0.873 | 0.873 | 0.873 | 0.873 |
|  |  | **Helioseal F (n = 5)** | **r_s_** | 0.200 | 0.200 | 0.200 | 0.308 | 0.359 |
|  |  |  | **p** | 0.747 | 0.747 | 0.747 | 0.614 | 0.553 |
|  | **Without Bond** | **Embrace WetBond (n = 5)** | **r_s_** | 0.400 | -0.900^*^ | -0.900^*^ | -0.900^*^ | -0.900^*^ |
|  |  |  | **p** | 0.505 | 0.037^*^ | 0.037^*^ | 0.037^*^ | 0.037^*^ |
|  |  | **Denuseal (n = 5)** | **r_s_** | 0.500 | 0.300 | -0.300 | -1.000^*^ | -0.200 |
|  |  |  | **p** | 0.391 | 0.624 | 0.624 | <0.001^*^ | 0.747 |
|  |  | **Helioseal F (n = 5)** | **r_s_** | -0.300 | -0.800 | -0.500 | -0.564 | -0.200 |
|  |  |  | **p** | 0.624 | 0.104 | 0.391 | 0.322 | 0.747 |
| **Sealant penetration** | **With Bond** | **Embrace WetBond (n = 5)** | **r_s_** | 0.300 | -0.500 | 0.000 | 0.700 | 0.700 |
|  |  |  | **p** | 0.624 | 0.391 | 1.000 | 0.188 | 0.188 |
|  |  | **Denuseal (n = 5)** | **r_s_** | -0.400 | -0.400 | -0.400 | -0.400 | -0.400 |
|  |  |  | **p** | 0.505 | 0.505 | 0.505 | 0.505 | 0.505 |
|  |  | **Helioseal F (n = 5)** | **r_s_** | 0.300 | 0.300 | 0.300 | 0.205 | 0.410 |
|  |  |  | **p** | 0.624 | 0.624 | 0.624 | 0.741 | 0.493 |
|  | **Without Bond** | **Embrace WetBond (n = 5)** | **r_s_** | -0.300 | -0.700 | -0.700 | -0.700 | -0.700 |
|  |  |  | **p** | 0.624 | 0.188 | 0.188 | 0.188 | 0.188 |
|  |  | **Denuseal (n = 5)** | **r_s_** | 0.300 | -0.200 | -0.300 | 0.300 | -0.800 |
|  |  |  | **p** | 0.624 | 0.747 | 0.624 | 0.624 | 0.104 |
|  |  | **Helioseal F (n = 5)** | **r_s_** | 0.100 | 0.400 | 0.300 | 0.051 | 0.600 |
|  |  |  | **p** | 0.873 | 0.505 | 0.624 | 0.935 | 0.285 |

**r_s_: Spearman coefficient**

*Statistically significant at p ≤ 0.05.
