# Supplementary material for: Microleakage, penetration depth, and fluoride release of Embrace Wetbond, Denuseal, and Helioseal F Plus pit and fissure sealants: a comparative in vitro study
Source: BMC Oral Health. 2026 May 6;26:796. doi: 10.1186/s12903-026-08419-y (PMC13151319; doi:10.1186/s12903-026-08419-y)
Supplement: Supplementary file 2 — Supplementary Material 2. [file 12903_2026_8419_MOESM2_ESM.pdf]

ATP Document Translation Services
University Development Center
Mansoura University

Certificate in Editing and Proofreading

This is to certify that the manuscript listed below was proofread and edited by Authorship, Translation & Publication (ATP), as confirmed by our signature below.

Manuscript title

Comparative Evaluation of Microleakage, Sealant Penetration, and Fluoride Release in Embrace WetBond, Helioseal F Plus, and Denuseal: An In Vitro Study

Authors

Heba Mohamed Mahmoud Deghid,Rabaa Mahmoud Aboubakr Hassan,Rasha Ibrahim Ramadan Saleh,Basma Elsayed Hamza Elboraey

Corresponding Author:
Heba Mohamed Mahmoud Deghid
Assistant Lecturer of Dental Public Health and Preventive Dentistry.
Department of Pediatric Dentistry and Dental Public Health.
Faculty of Dentistry, Mansoura University, Mansoura City, Egypt.
Email: heba.mohamed@mans.edu.eg

Affiliation

Assistant Lecturer of Dental Public Health and Preventive Dentistry, Department of Pediatric Dentistry and Dental Public Health. Faculty of Dentistry, Mansoura University, Mansoura City, Egypt.
Professor of Dental Public Health and Preventive Dentistry, Department of Pediatric Dentistry and Dental Public Health. Faculty of Dentistry, Mansoura University, Mansoura City, Egypt.
Assistant Professor of Dental Public Health and Preventive Dentistry, Department of Pediatric Dentistry and Dental Public Health. Faculty of Dentistry, Mansoura University, Mansoura City, Egypt.
Lecturer of Dental Public Health and Preventive Dentistry, Department of Pediatric Dentistry and Dental Public Health. Faculty of Dentistry, Mansoura University, Mansoura City, Egypt.

مركز التطوير الجامعي
University Development Center
Date
13/1/2026
Certificate number
2000

Dr. Leena Seif	Dr. Eman Ebeida	Assoc. Prof. Mohamed Abdelkhalek	Dr. Mohamed A. Ghonim
leena Seif	[illegible]	Mohamed Abd	M. Ghon
Proofreader	ATP Director	UDC Deputy Director	UDC Director

CamScanner
